# Validation and cost-effectiveness of an mHealth tool for cognitive impairment detection in Peru: protocol for the IMPACT Salud observational study

**DOI:** 10.1136/bmjopen-2025-107142

**Published:** 2025-11-09

**Authors:** Maria Kathia Cardenas, Cecilia Anza-Ramirez, Antonio Bernabe-Ortiz, Nilton Custodio, Rosa Montesinos, Juan Jaime Miranda, Marco Da Re, Maria Veronica Belon-Hercilla, Maria Lazo-Porras, Jemma Hawkins, Francisco Diez-Canseco, Graham Moore, William Whiteley, Rafael A Calvo, Maria Sofia Cuba-Fuentes, Filipa Landeiro, Christopher R Butler, On behalf of the IMPACT Salud Study Group

**Affiliations:** 1 Nuffield Department of Population Health, Health Economics Research Centre, University of Oxford, Oxford, UK; 2 CRONICAS Center of Excellence in Chronic Diseases, Universidad Peruana Cayetano Heredia, Lima, Peru; 3 Department of Brain Sciences, Imperial College London, London, UK; 4 Unidad de Diagnóstico de Deterioro Cognitivo y Prevención de Demencia, Instituto Peruano de Neurociencias, Lima, Peru; 5 Unidad de Investigación, Instituto Peruano de Neurociencias, Lima, Peru; 6 Sydney School of Public Health, Faculty of Medicine and Health, University of Sydney, Camperdown, New South Wales, Australia; 7 Dyson School of Design Engineering, Imperial College London, London, UK; 8 School of Social Sciences, Centre for Development, Evaluation, Complexity and Implementation in Public Health Improvement (DECIPHer), Cardiff University, Cardiff, UK; 9 Centre for Clinical Brain Sciences, The University of Edinburgh, Edinburgh, UK; 10 Center for Research in Primary Health Care, Universidad Peruana Cayetano Heredia, Lima, Peru; 11 The George Institute for Global Health, School of Public Health, Imperial College London, London, UK

**Keywords:** Dementia, HEALTH ECONOMICS, Quality of Life, Aging, EPIDEMIOLOGY

## Abstract

**Abstract:**

**Introduction:**

Dementia is a chronic and progressive neurological condition characterised by cognitive and functional impairment. It is often associated with multimorbidity and imposes a significant economic burden on healthcare systems and families, especially in low-income and middle-income countries. In Peru, where dementia cases are increasing rapidly, timely detection and referral for diagnosis is crucial. This protocol is part of the IMPACT Salud project in Peru. Here, we focus on a specific component aimed at validating an mHealth tool for the detection of cognitive and functional impairment and assessing its cost-effectiveness. We will also assess changes in cognitive and functional impairment as well as health economic outcomes over 1 year.

**Methods and analysis:**

This observational study will be conducted in four geographically diverse regions of Peru. Community health workers are expected to contact approximately 32 000 participants (≥60 years) to apply an mHealth-enabled tool that includes cognitive and functional instruments: Ascertain Dementia 8, Peruvian version of Rowland Universal Dementia Assessment Scale and Pfeffer Functional Activities Questionnaire. From this large sample, we aim to find 3600 participants and their study partners to enrol and interview at baseline regarding sociodemographic characteristics, lifestyles, comorbidities and health economic data including resource use, costs and health-related quality of life (HR-QoL). Psychologists, blind to previous results, will assess dementia stage of the participants using an abbreviated Clinical Dementia Rating (CDR) scale. At 6-month follow-up, participants will complete a brief health economics questionnaire on resource use, costs and HR-QoL. To validate the accuracy of the detection tool, a subsample of 600 participants who completed the baseline will undergo a gold-standard clinical neuropsychological assessment. This subsample will participate in a 12-month follow-up, including health economics, cognitive and functional impairment tests and the CDR scale. Results will be analysed and presented by cognitive status, site, sex and multimorbidity profile. Finally, data from all stages and external sources will inform a decision model to implement a cost-effectiveness analysis of the detection tool at the national level.

**Ethics and dissemination:**

The study received ethics approval in Peru (Universidad Peruana Cayetano Heredia: CONSTANCIA-CIEI-378-33-23) and in the UK (Imperial College London: ICREC/SETREC reference number 6647445). Informed consent will be obtained from participants and their study partners, considering the participant’s capacity to consent. For illiterate participants, consent will be obtained through a witnessed procedure involving study partners, with a fingerprint obtained instead of a signature. The results will be disseminated through conferences, published articles, public presentations (particularly to those involved in dementia care) and presentations or meetings with local health authorities.

**Strengths and limitations of this study**

- This study will apply appropriate tests for cognitive and functional impairment considering the cultural and educational differences in Peru.
- Cognitive and functional impairment tests will be applied using an mHealth tool by trained community health workers in settings where specialists are scarce.
- This study will comprehensively estimate the economic burden and health-related quality of life associated with dementia in Peru, using a societal perspective and a longitudinal design.
- Limited prior knowledge of technology and mental health among community health workers presents a challenge.
- Self-reported resource use and costs rely on a 6-month recall period; however, mitigation strategies will be implemented.

## Introduction

 Dementia is a chronic and progressive neurological condition characterised by cognitive decline and impairment of daily functioning,^1^ and it is often associated with multimorbidity.^2 3^ In low-income and middle-income countries (LMICs), where an estimated 60% of people with dementia reside,^4^ health systems are insufficiently equipped to diagnose and manage the rising burden of dementia.^5^,^7^ In Peru, dementia cases are expected to triple by 2050,^8^ which highlights the need for accessible and effective screening, especially for socioeconomically disadvantaged populations. Developing and validating appropriate tools for cognitive and functional impairment is crucial to reduce bias related to literacy, education and cultural factors.^9 10^ The growing number of people living with dementia imposes a significant economic burden on societies, with costs increasing rapidly as the disease progresses to more advanced stages.

Diagnosing dementia in LMICs is challenging due to low awareness, stigma, limited healthcare access and inadequate training of health professionals.^56 11^,^13^ Moreover, dementia care heavily relies on family members, imposing a high socioeconomic burden on caregivers.^14^,^16^ A reliable screening for dementia, delivered by non-specialists as recommended by the WHO,^17^ and adaptable across multiple contexts, could reduce barriers to timely diagnosis and enhance care planning.

In response to these challenges, the IMPACT Salud project was developed to strengthen Peru’s health system using dementia as a tracer condition.^18^ The project comprises five work packages (WP), each with a different aim: WP 1 assesses health system readiness for dementia; WP 2 designs, validates and implements the IMPACT Salud App for cognitive and functional impairment detection; WP 3 adapts and assesses the feasibility of an mHealth intervention for people with dementia and their caregivers; WP 4 estimates the economic burden and impact on health-related quality of life (HR-QoL) of dementia and assesses the cost-effectiveness of the detection tool and WP 5 coordinates project management as well as community engagement and involvement. This observational study protocol is only about the work carried out by WP 2 and WP 4.

The overarching aim of this study is to validate an mHealth-enabled detection tool for cognitive and functional impairment in Peru, and to assess its cost-effectiveness. Additionally, it will assess changes in health economic outcomes over 1 year in relation to the progression of the disease. The specific objectives are:

- To assess the accuracy of an mHealth-enabled tool for cognitive and functional impairment detection.
- To characterise clusters of comorbidities across different stages of dementia and identify associated factors.
- To estimate the economic burden and the impact on HR-QoL of dementia in Peru, considering dementia stage and chronic comorbidities.
- To assess longitudinal changes in resource use, costs, HR-QoL and cognitive impairment status over 1 year.
- To determine the costs and cost-effectiveness of the mHealth-enabled tool in the Peruvian health system.

## Methods and analysis

Prior to this observational study, extensive work was carried out to design, develop and field-test an mHealth-enabled tool for cognitive and functional impairment detection, as detailed elsewhere.^19 20^ In summary, the system comprises a technological component to apply tests via tablet, alongside a human component, involving trained community health workers (CHWs) responsible for conducting door-to-door visits. This section outlines the observational study jointly conducted by WP 2 and WP 4 of the IMPACT Salud project. Following completion of the study, and on validation of the system, we will provide insights into its potential integration into selected primary healthcare centres (PHCs) in Peru. The implementation within PHC falls outside the scope of this observational study protocol; detailed description of this component will be addressed in a subsequent publication, alongside a process evaluation to assess implementation outcomes.

### Study design

**Figure 1:**
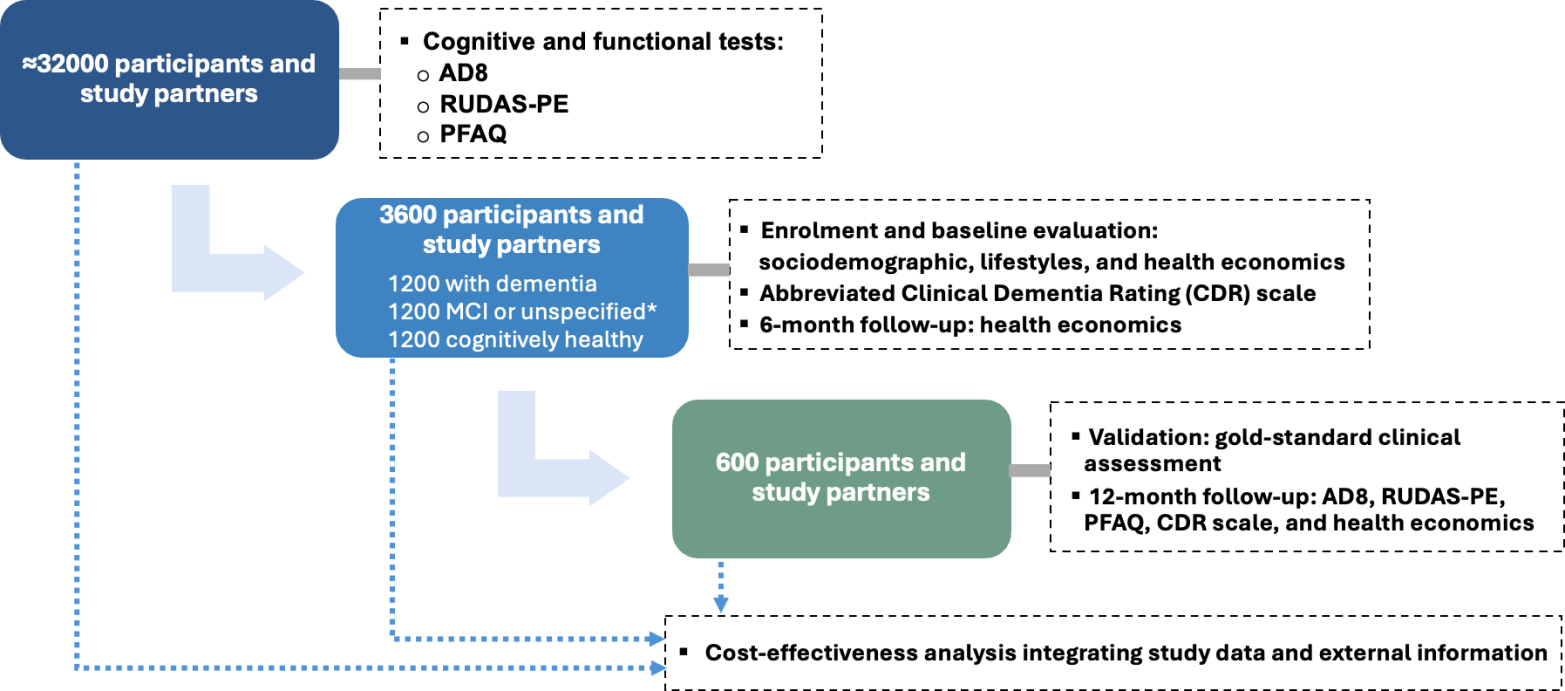
Design of the study. *Participants impaired on only one cognitive assessment were classified as having unspecified cognitive impairment. AD8, Ascertain Dementia 8; MCI, mild cognitive impairment; PFAQ, Pfeffer Functional Activities Questionnaire; RUDAS-PE, Peruvian version of Rowland Universal Dementia Assessment Scale.

This observational study follows a multistage approach (figure 1), consisting of (1) a cross-sectional cognitive and functional impairment assessment of ~32 000 participants delivered by CHWs, who act as a bridge between the community and PHC services, using a tablet with an mHealth-enabled detection tool; (2) a cross-sectional baseline evaluation of sociodemographic characteristics, lifestyles, comorbidities, health economic outcomes (resource utilisation, costs, and HR-QoL) and dementia stage in a sample of individuals (3600 participants and study partners); (3) a longitudinal evaluation of cognitive and functional impairment, and health economic outcomes in a subsample of 600 participants and study partners; (4) validation of the mHealth-enabled detection tool against a gold-standard clinical assessment in the subsample of 600 participants and study partners and (5) cost-effectiveness analysis of the detection tool using information from previous stages.

### Study setting 

The IMPACT Salud project is being conducted in Peru, an upper-middle-income country in South America with diverse geographical, socioeconomic conditions and cultural diversity. The project includes four sites representing this diversity: Lima, the capital city, is highly urbanised and home to one-third of the population; Tumbes, a semiurban area on the northern coast; Huancayo, a city located at 3200 m above sea level in the central Andes; and Iquitos, the largest city in the Peruvian Amazon.

### Participants and recruitment

Eligible participants must be older adults (60 years or older) and full-time residents of the study’s settings, having lived there for at least 6 months prior to enrolment. Each participant must have a study partner, defined as an individual 18 years or older who can provide information about the participant’s health. Only participants who are able to identify a study partner will be enrolled. Participants and study partners will be recruited through door-to-door visits conducted by CHWs.

### Sample size

The estimated total sample of approximately 32 000 participants (8000 per site) for the detection of cognitive and functional impairment was determined to ensure finding 1200 individuals with possible dementia and 1200 cognitively healthy. An additional 1200 participants classified as having possible mild cognitive impairment (MCI) or placed in an unspecified category of cognitive impairment will form a third group. These 3600 participants will be included in a subsequent cross-sectional study. Then, 600 will participate in the longitudinal study and validation process.

The subsample of 3600 participants was calculated using EPIDAT V.4.2 software. This sample size provides ≥80% power to detect a difference of at least 7% in the prevalence of any morbidity or sociodemographic characteristic between groups (with dementia and without dementia) at a 95% confidence level. With this sample size, the study will achieve a precision of at least 2.5% to detect a sensitivity ≥75% for the detection system, assuming a disease/non-disease ratio of 1, a 95% confidence level, and a specificity ranging from 50% to 100%.

From the 3600 participants, a subsample of 600 participants will be randomly selected for gold-standard validation, comprising 200 participants with dementia, 200 with MCI and 200 controls (cognitively healthy). This sample size ensures a precision of 6% to detect a sensitivity of at least 75%, assuming a disease to non-disease ratio of 1, a 95% confidence level, and a specificity varying from 50% to 100%. These 600 participants will also be included in the 12-month follow-up to assess changes in cognitive and functional impairment and health economic outcomes.

### Procedures and data collection tools

This study begins with the detection of cognitive and functional impairment, followed by a baseline evaluation and short 6-month follow-up. A further subsample will undergo clinical validation of the detection tool and participate in a 12-month follow-up. The findings from all stages will be integrated into a cost-effectiveness analysis. Prior to starting the fieldwork, CHWs completed a 2-week training programme. Fieldwork began in September 2024 and is expected to be completed by September 2026.

#### Cognitive and functional impairment detection

CHWs will conduct door-to-door visits targeting approximately 32 000 participants (8000 per site).^20^ The cognitive and functional tests included are: (1) the Ascertain Dementia 8 (AD8),^21^ reported by the study partner (eg, caregiver or relative); (2) the Peruvian version of Rowland Universal Dementia Assessment Scale (RUDAS-PE) administered to the participants^22 23^ and (3) a functional assessment using the Pfeffer Functional Activities Questionnaire (PFAQ), reported by the study partner.^24 25^

Participants will first be assessed using the AD8 and RUDAS-PE. A positive detection result is defined as an AD8 score of ≥2 and a RUDAS-PE score of <19 for illiterate participants or <21 for literate participants, while a negative result is any score outside these thresholds.^21^,^23^ Those who test negative on AD8 and RUDAS-PE will be considered cognitively healthy, while those with positive results to both will be classified as having cognitive impairment and will proceed to the PFAQ test.^21^,^23^ Participants with a PFAQ score of ≥7 will be classified into the dementia group, while those scoring <7 will be in the MCI group.^24 25^ Finally, unspecified cases—defined as those with discordant results (AD8 positive but RUDAS-PE negative or vice versa)—will be classified during the next phase, which includes dementia staging using the abbreviated Clinical Dementia Rating (CDR) scale only for participants enrolled in the baseline evaluation. Table 1 presents a description of each test.

**Table 1:** Description of cognitive and functional tests implemented by community health workers

Test	Description	Possible results
AD8	Brief instrument composed of 8 questions. It helps screen for early cognitive changes related to dementia from the perspective of someone close to the participant.	For each question, 1 point is awarded if there is a change and 0 points if there is not. If the person does not know, a third option that does not give numerical value is marked. The maximum score is 8; the higher the score, the greater the cognitive impairment. If the sum of the score is 2 or more, it is considered a positive test result (suggestive of cognitive impairment); if the score is less than 2, the participant may have normal cognitive function.
RUDAS-PE	Brief cognitive screening assessment designed to reduce the impact of cultural and linguistic context on the initial assessment of cognitive performance. It helps to identify individuals with dementia from those without dementia. It consists of 6 tasks given to the participant to test: recent memory, body orientation, motor praxis, visuospatial construction, judgement (abstract reasoning and problem solving) and language (semantic verbal fluency).	Each task has a differentiated score. Those with a RUDAS-PE score <19 points (when the participant is illiterate) or <21 points (otherwise) will be considered positive (potential dementia or MCI).
PFAQ	This functional test complements the cognitive assessment with information provided by the study partner. The test has 11 questions to assess participants’ ability to perform instrumental activities of daily living.	Each question is scored from 0 to 3 points, where 0 represents low or no need of help/assistance and 3 represents high dependency on help/assistance. A score >6 indicates functional impairment.

AD8, Ascertain Dementia 8; MCI, mild cognitive impairment; PFAQ, Pfeffer Functional Activities Questionnaire; RUDAS-PE, Peruvian version of Rowland Universal Dementia Assessment Scale.

#### Baseline evaluation on the 3600 participants’ subsample

In total, 3600 participants and their study partners (900 per site) will be enrolled in the baseline evaluation, including 1200 participants with possible dementia based on the cognitive and functional impairment results, 1200 with possible MCI or classified as unspecified, and 1200 cognitively healthy individuals. Participants will be enrolled into the baseline study in the order they are visited, while simultaneously maintaining balance in the number of recruited participants across the three groups. CHWs will conduct face-to-face interviews covering three specific domains: sociodemographic and lifestyle factors, comorbidities and health economic data (including resource use and HR-QoL). Information about the domains and subdomains assessed is presented in table 2.

**Table 2:** Domains and subdomains covered in baseline and follow-up assessments, by respondent type

Domain	Subdomain	Respondent		Assessments		
		Participant	Study partner	B	FU-6 m	FU-12
Sociodemographic and lifestyles	Demographic information	X	X	X		
	Household assets	X		X		
	Health coverage (insurance)	X	X	X		
	Technological devices and internet access	X		X		
	Speech and language markers^59^	X	X	X		
	Tobacco use	X		X		
	Alcohol use	X		X		
	Physical activity/inactivity	X		X		
	Sleep	X		X		
Comorbidities	Depressive symptoms	X	X	X		
	Anxiety	X		X		
	Health background (doctor-diagnosed conditions)	X	X	X		
	Falls	X		X		
	Height and weight	X		X		
	Blood pressure	X		X		
	Visual Acuity (Snellen Test)	X		X		
	Hearing (Pure tone test)	X		X		
	10 m walk test	X		X		
	Grip strength	X		X		
	Frailty	X		X		
	Zarit burden interview		X	X		X
Health economics	EuroQol 5-dimension, 5-level instrument (EQ-5D-5L self-rated quality of life, face-to-face)	X	X	X		X
	EuroQol 5-dimension, 5-level instrument (EQ-5D-5L proxy 1 version, face-to-face)		X	X		X
	EuroQol 5-dimension, 5-level instrument (EQ-5D-5L self-rated quality of life, telephone version)	X			X	
	Work status and usual activities	X	X	X		X
	Resource utilisation and costs (full version)	X	X	X		X
	Resource utilisation and costs (short version)	X			X	
Dementia staging (dementia severity)	Abbreviated CDR scale (applied by neuropsychologists)	X	X	X		X

B, baseline; CDR, Clinical Dementia Rating; F-6 m, follow-up at 6 months; F-12 m, follow-up at 12 months.

Participants and their study partners will provide information about age, sex, education level, ethnicity proxy (using language and self-identity), and other characteristics. Lifestyle factors focus on participants’ behaviours associated with cognitive impairment, including smoking, alcohol consumption, sleep and physical activity.

Comorbidities and other health conditions will be evaluated using objective measures following standard guidelines, as detailed in table 3, as well as through self-reported data.^26^,^36^ In participants, the evaluation includes hypertension, type 2 diabetes mellitus, depression, anxiety, hearing impairment (deafness), visual acuity (blindness), mobility limitations, grip strength, overweight/obesity, self-reported falls and frailty. Participants and their study partners will also provide self-reported data on doctor-diagnosed conditions (health background). Additionally, caregiver burden and depression will be assessed in study partners.

**Table 3:** Comorbidities or conditions assessment

Comorbidity/condition	Evaluation tool	Guidelines or description
Hypertension	Self-report of history of doctor diagnosis. Three blood pressure measurements after a 5 min rest, with measurements separated by at least 1 min	International recommendations.^26^ Taken by trained CHWs using an automatic blood pressure monitor
Type 2 diabetes mellitus	Self-report of doctor-diagnosis Blood sample after an 8–12-hour fasting period (fasting glucose measurement)	American Diabetes Association (fasting glucose)^27^ Blood glucose sampling process by trained laboratory staff
Depression	Patient Health Questionnaire-9 (PHQ-9)	Previously used in Peru^28^,^30^
Anxiety	General Anxiety Disorder-7 (GAD-7)	Validated in Spanish in Colombia and Peru^31 32^
Hearing	Simple audiometry test (Pure tone test)	Standard audiometry practices
Visual acuity	Snellen chart test	Standard visual acuity assessment
Mobility	Walking test (10 m walk)	Standard mobility assessment
Grip strength	Hand grip strength test	A hand dynamometer will be used to measure grip strength
Other chronic conditions (self-report)	Self-reported checklist of doctor-diagnosed conditions, including dementia, Parkinson’s, hypertension, diabetes, cholesterol, chronic obstructive pulmonary disease, angina/heart attack/myocardial infarction, stroke, acid peptic disease/gastritis, arthritis, cancer, chronic kidney disease, epilepsy/convulsion, osteoporosis, thyroid disease, chronic back pain, joint pain or inflammation, chest pain, paralysis or weakness on one side	A question similar to that used in the Multimorbidity Assessment Questionnaire for Primary Care (MAQ-PC) tool^33^: ‘Have you ever been diagnosed by a doctor with (condition)?’ However, we have included additional conditions and have not used all questions from the MAQ-PC
Frailty	Social Frailty Index and Clinical Frailty Scale questionnaire (short version)	The Social Frailty Index comprises questions about social risk factors, including age, gender, neighborhood cleanliness, financial control, volunteer and paid work, social and family engagement, feelings of isolation, and lack of respect.^34^ The Clinical Frailty Scale questionnaire (short version) comprises 7 items assessing overall frailty, including overall health, chronic conditions, assistance required for basic and instrumental activities of daily living, effort required, and physical activities^34 35^
Body mass index	Weight and size assessment	A mobile wooden measuring rod will be used for height measurement and a Seca floor scale model 813—CE with a super wide platform will be used for weight measurement
Caregiver burden	Zarit burden interview	Previously used in Peru^36^

CHWs, community health workers.

Both participants and their study partners will be interviewed to assess their resource use and HR-QoL. To collect data on resource use, a questionnaire from the Health Economics of Pre-dementia Alzheimer’s Disease: Pilot Study^37^ was translated to Spanish and adapted to the Peruvian context. Adaptation began with a desk review and was followed by discussions with CHWs, a clinician and a caregiver representative, during which the questionnaire was reviewed and further modifications were made as needed. The questionnaire will capture resource use and out-of-pocket expenses on hospitalisations, emergency visits, outpatient consultations, medications, medical tests, formal care and other resources used in relation to their condition. It will also assess hours of informal care provided by study partners and other caregivers and productivity losses due to work absenteeism of both the participants and study partners. HR-QoL will be assessed using the EuroQol 5-dimension, 5-level instrument (EQ-5D-5L).^38 39^ The self-rated version will be administered to participants and their study partners to obtain information about their own health status, while the proxy-1 version will be completed only by study partners to obtain information about the participant’s HR-QoL.

#### Dementia staging in the 3600 participants’ subsample

For the baseline evaluation of the dementia severity, participants and their study partners will be interviewed by a neuropsychologist, who will administer the abbreviated CDR scale.^40 41^ The neuropsychologists from the Peruvian Institute of Neurosciences (Instituto Peruano de Neurociencias) will be blinded to participants’ prior cognitive results. All participants will be categorised into three groups: cognitively healthy (CDR score of 0), MCI (CDR score of 0.5) and dementia (CDR score greater than 0.5). Within the dementia group, stages will be further classified as mild dementia (CDR score of 1), moderate dementia (CDR score of 2) and severe dementia (CDR score of 3). At this stage, participants initially classified as ‘unspecified’ will be reassigned to a specific cognitive impairment category.

#### 6-month follow-up: health economics data collection in the 3600 participants’ subsample

6 months after baseline, the 3600 participants (assisted by their study partners, if needed) will be interviewed by telephone to assess health economic outcomes such as hospitalisation admissions, emergency visits, outpatient consultations, medication, exams, formal care, informal caregiving hours and out-of-pocket expenses. Additionally, participants will respond to the self-rated telephone version of the EQ-5D-5L.

#### Validation of the mHealth-enabled detection tool against gold standard in the 600 participants’ subsample

From the 3600 participants enrolled, a subsample of 600 participants and their study partners (150 per study site) will be selected to participate in the validation phase using a comprehensive clinical assessment considered as the gold standard for diagnosing dementia. The assessment will be conducted by a team of clinicians and neuropsychologists from the Peruvian Institute of Neurosciences and includes: a neuropsychological evaluation based on the Uniform Data Set from the National Alzheimer’s Coordinating Center-NACC (UDS, NB 3.0),^42 43^ social cognition using the mini Social Cognition and Emotional Assessment,^44 45^ neuropsychiatric symptoms using the Neuropsychiatric Inventory,^46^ functionality assessment through the Technology–Activities of Daily Living Questionnaire,^47^ and the CDR scale.^40 41^ The final classification of MCI and dementia will follow the criteria of the Diagnostic and Statistical Manual of Mental Disorders, Fifth Edition (DSM-5)^1^ criteria through a consensus among the team, while severity of dementia will be categorised according to the CDR scale.

#### 12-month follow-up: cognitive and functional impairment assessment, dementia staging and health economics data

12 months after baseline, the 600 participants in the validation subsample will be followed up through face-to-face interviews to evaluate changes in cognitive and functional impairment and health economic outcomes. Cognitive and functional changes will be assessed using the AD8, RUDAS-PE, PFAQ and the CDR scale. The health economic outcomes include resource use, costs and HR-QoL reported by both participants and their study partners.

#### Cost-effectiveness analysis

We will conduct a cost-effectiveness analysis of the mHealth-enabled tool using a decision analytic model from a healthcare perspective. Model structure will be defined following the recommendations provided by experts in the field of dementia regarding decision-analytic modelling.^48^ The model will include data on the sensitivity and specificity of the mHealth-enabled detection tool for cognitive and functional impairment obtained from the detection and validation stages of the study. We will collect information on the resources and costs of implementing the detection tool using a data collection form. The implementation costs will include training of CHWs, the development of the mHealth-enabled tool, equipment costs (eg, tables) and internet connection, door-to-door visits to reach participants, confirmatory evaluation with clinical assessment and follow-up medical consultations. The model will incorporate data on healthcare costs collected from study participants and valued using unit prices from the Peruvian Ministry of Health to capture clinical pathways after diagnosis (eg, referral to specialised services). Additionally, external data on the national sociodemographic distribution will be gathered from publicly available datasets from the Peruvian statistics office (INEI), while the proportion of participants with dementia and their characteristics by cognitive impairment status will be gathered from study findings and existing literature.

### Statistical analysis

This section outlines the analysis plan for the study outcomes listed in table 4.

**Table 4:** List of study outcomes

Domain	Outcomes
Cognitive and functional impairment	Completion rate of cognitive and functional impairment assessment: The percentage of participants who completed the cognitive and functional impairment assessment relative to total eligible population. Sensitivity and specificity of the detection tool: Validation of AD8, RUDAS-PE and PFAQ against the gold-standard clinical assessment to determine their effectiveness in detecting possible dementia. Classification accuracy: The proportion of correctly classified cases with dementia and without dementia when compared with the final clinical assessment diagnosis. Proportion of cognitive impairment status: The proportion of participants by cognitive impairment status (cognitively healthy, MCI, dementia) across the four study sites by sex. Domain/question specific analysis: Analysis of the most frequently impaired domain or specific question in each cognitive and functional test, categorised by classification group (control, unspecified, MCI and dementia), study site and sex. Follow-up outcomes on cognitive impairment cases: Changes in cognitive impairment among the validation subsample (600 participants) after approximately 12 months. Demographic and geographic distribution of cognitive impairment cases: Identification of variations in cognitive impairment cases based on age, sex, education level and study site.
Comorbidities	Proportion of clinically assessed (objective measure) chronic conditions at baseline: The proportion of participants diagnosed with cardiovascular diseases (hypertension, stroke, heart disease), metabolic disorders (type 2 diabetes, obesity), mental health conditions (depression, anxiety), sensory impairments (hearing loss, visual impairment), frailty and mobility limitations. Proportion of other self-reported chronic conditions at baseline: Self-report conditions including arthritis, chronic lung disease, acid peptic disease, chronic back pain, heart disease, stroke, alcohol disorder, cancer, chronic kidney disease, epilepsy, thyroid disease and tuberculosis will also be described for the participants and their study partner. Multimorbidity patterns at baseline: The co-occurrence of two or more chronic conditions among participants. Health disparities at baseline: Variations in chronic condition proportions and multimorbidity patterns across study sites and demographic subgroups. Impact of comorbidities on cognitive function at baseline: The relationship between chronic conditions and cognitive impairment severity.
Health economics and cost-effectiveness	Resource use in participants and their study partners by cognitive impairment status: Annual resource use of emergency visits, hospitalisations, outpatient consultations, medication use, diagnostic and laboratory tests, and hours of formal and informal care. Costs in participants and their study partners by cognitive impairment status and multimorbidity pattern: Annual total cost defined as the sum of annual healthcare and non-healthcare costs, informal care cost and productivity loss. Annual total costs of participants by cognitive impairment status and with different number of comorbidities. Changes in costs by cognitive impairment status after 12 months compared with baseline: Variation after 12 months in estimated costs for participants, study partners and the dyad comparing groups with different levels of cognitive and functional impairment. HR-QoL in participants and their study partners by cognitive impairment status and multimorbidity at baseline and follow-up (6 month and 12 month): EuroQol 5-dimension, 5-level instrument (EQ-5D-5L), EQ-5D-5L index, and EuroQol-visual analogue scale (EQ VAS) scores of self-reported and proxy-rated versions comparing groups with different levels of cognitive and functional impairment and with different number of comorbidities. Changes in HR-QoL in participants and their study partners at 12 months by cognitive impairment status: EQ-5D-5L dimensions, EQ-5D-5L index and EQ VAS scores of self-reported and proxy-rated versions comparing changes after 12 months across groups with different levels of cognitive and functional impairment. Cost per assessed participant: The cost of implementing the detection tool per individual who completed the assessment. Cost per correctly identified case of dementia: The cost of implementing the detection tool per individual accurately diagnosed with dementia. Incremental cost-effectiveness ratio: The additional cost of detecting and diagnosing dementia cases compared with current situation.

AD8, Ascertain Dementia 8; MCI, mild cognitive impairment; PFAQ, Pfeffer Functional Activities Questionnaire; RUDAS-PE, Peruvian version of Rowland Universal Dementia Assessment Scale.

#### Detection of cognitive and functional impairment 

We will assess cognitive and functional impairment as well as dementia severity using data collected at different time points and across subsamples. At baseline, among all participants, we will estimate the proportion classified as potentially having cognitive and functional impairment using the AD8, RUDAS-PE and PFAQ tools, stratified by study site and sex. We will also conduct domain-specific analyses to identify the most frequently impaired cognitive domains or questions within each tool and classification group. Using information from 3600 participants, we will determine the proportion of participants at each dementia stage and compare groups using descriptive statistics and statistical tests (eg, χ² test). To evaluate change over time, we will analyse longitudinal data from the follow-up subsample, 600 participants, assessed at baseline and again after approximately 12 months, using generalised linear or mixed models to estimate changes in cognitive impairment status and dementia severity, adjusting for sociodemographic characteristics and multimorbidity. Finally, in this same subsample, we will assess the sensitivity and specificity of the AD8, RUDAS-PE and PFAQ tools against a clinical gold-standard diagnosis to evaluate their accuracy in detecting possible dementia. Sensitivity will be defined as the proportion of true dementia cases correctly classified, and specificity as the proportion of non-dementia cases correctly identified.

#### Comorbidities

Using data from the baseline evaluation, we will calculate proportions and other descriptive statistics for both objective measures and self-reported data on chronic conditions, including cardiovascular, metabolic, mental health and sensory disorders, as well as frailty and mobility limitations among participants. We will analyse multimorbidity patterns and health disparities by dementia stage, across study sites and demographic subgroups, using appropriate statistical methods. Furthermore, we will assess the impact of chronic conditions and multimorbidity on cognitive function, exploring their association with cognitive impairment severity. Multivariable regression models will be used to estimate these associations, adjusting for sociodemographic characteristics.

#### Health economics: resource utilisation, costs, HR-QoL and cost-effectiveness analysis

We will estimate the total annual cost for participants, their study partners and the dyad by cognitive impairment status using baseline evaluation data from 3600 participants related to direct costs, informal care and productivity costs. Self-reported resource use data related to direct costs over 6 months will be annualised and multiplied by unit costs obtained from publicly available sources from the Peruvian Ministry of Health. Informal care costs will be estimated by annualising caregiving hours and valuing them using two methods: (1) the opportunity cost method, which values caregiver time using the average wage in their employment sector or the minimum wage if unemployed and (2) the replacement cost method, which uses the market price of formal care as a proxy. Productivity losses will also be annualised and valued by estimating foregone earnings using the average wage of the respondent’s employment sector. A second scenario of the annual cost estimation will incorporate data from the 6-month follow-up, providing a refined estimation. We will estimate mean and SE of resource use and costs. Costs will be compared across cognitive impairment status groups (cognitively healthy, MCI and dementia stages: mild, moderate and severe) and considering additional comorbidities. Generalised linear models (eg, Gamma GLM with log link) will be used to assess cost drivers such as chronic comorbidities, adjusting for age, sex, socioeconomic status, marital status and region.

To estimate the economic burden at the national level, we will consider uncertainty using probabilistic sensitivity analysis in key cost variables (eg, hospitalisations, caregiving time, unit costs) and different scenarios. These costs will then be scaled up to a national level using national statistics of the Peruvian population (population by sex and age group), and data on the prevalence of dementia and disease stage to determine the annual full economic cost of the disease.

We will also assess changes in costs of dementia and its association with key cost drivers, including dementia severity, over 12 months in a subsample of 600 participants and their study partners. We will use panel data regression models with fixed and random effects to estimate changes in costs over 12 months and to identify key cost drivers.

We will analyse the results of applying the EQ-5D-5L at baseline, 6-month follow-up and 12 month follow-up using proportions of problems found in each of the five dimensions by age group and cognitive impairment status. The distribution across each level of EQ-5D-5L domains will be examined to identify the domains that were most affected by different groups. EuroQol-visual analogue scale (EQ VAS) scores will be analysed using measures of central tendency and dispersion. Health state utility values will be estimated using the EQ-5D-5L value set for Peru.^49^ We will analyse how the HR-QoL of people with dementia and study partners varies with dementia stage using regression models. We will also assess how HR-QoL is affected by changes in dementia stage in the subsample of 600 participants and study partners at 12 months using multiple regression model adjusted by baseline results, sociodemographic characteristics and chronic comorbidities using panel data regression models (fixed and random effects models).

In the cost-effectiveness analysis, we will estimate the cost per individual assessed, the cost per true positive diagnosis and the incremental cost-effectiveness ratios, such as the incremental cost per additional individual assessed and the incremental cost per additional case correctly diagnosed with dementia. Sensitivity analyses will be conducted to consider the uncertainty on different key input variables, including different health system constraints (eg, availability of trained workers to implement the detection tool, availability and cost of the confirmatory evaluation). We will perform both deterministic and probabilistic sensitivity analyses.

### Patient and public involvement

Our study involves the active participation of a caregiver representative, who also serves as a coinvestigator, from the initial design of the research proposal and throughout the project. This representative provides advice or feedback when needed and has also played a key role facilitating the contact with other patients and caregivers. In addition, dyads of patients and caregivers, as well as CHWs, provided direct input into the development of the IMPACT Salud app. The participation of patients, caregivers and CHWs has been described in detail elsewhere.^19 20^ The caregiver representative and CHWs also contributed feedback on the adaptation of the resource utilisation questionnaire through individual online working sessions, ensuring that a range of ideas and perspectives were incorporated. We plan to continue seeking input and feedback from both the caregiver representative and CHWs throughout the project and until the dissemination phase.

### Ethics and dissemination

This project has received ethical approval from the Institutional Review Board at Universidad Peruana Cayetano Heredia in Peru (SIDISI Code: 209080), and the Imperial College Research Ethics Committee (ICREC reference number: 6647445) in the UK. Informed consent will be obtained from participants and their study partners. For participants who are illiterate, informed consent will be obtained using a witnessed procedure, in this case, the study partner. Participants will be invited to provide their fingerprint in place of a signature.

For individuals with cognitive impairment, the decision regarding participation will be based on an assessment of the participant’s capacity to consent. If the participant is determined to lack capacity, the study partner will be asked to provide consent on their behalf. Fieldworkers will explain the study’s objectives, benefits and risks, provide additional information if requested and allow time for questions before obtaining written consent.

This study has minimal risk for participants; however, a log for adverse events will be maintained. Risks associated with blood sampling (eg, discomfort, minor pain, bruising or vasovagal reactions) and potential stress at finding out they have cognitive impairment or a chronic condition (eg, hypertension, type 2 diabetes) will be clearly communicated. All serious adverse events will be reported to the Ethics and Research Governance Coordinator. Each participant will be assigned a unique identification code to ensure confidentiality, and data will be securely stored in password-protected electronic systems with restricted access to identifiable information. No monetary reimbursement will be provided, but participants may benefit from counselling and diagnosis related to cognitive and functional impairment, hypertension or diabetes.

The results will be disseminated through conferences, published articles, public presentations (particularly to those involved in dementia care), and presentations or meetings with local health authorities.

## Discussion

This study will be the first in Latin America to evaluate the validation and cost-effectiveness of a community-based mHealth-enabled tool for cognitive and functional impairment detection delivered by CHWs. The technology-enabled system includes tests appropriate for diverse educational backgrounds and resource-limited settings, such as AD8, RUDAS-PE and PFAQ, all administered using a tablet. Engaging CHWs as key implementers of the detection process is particularly important to enhance reach, trust and participation. CHWs have been defined by the Peruvian Law No. 30 825 as individuals selected by their communities to voluntarily collaborate with healthcare workers and local organisations in tasks related to prevention and health promotion.^50^ This study will be the first to estimate the economic burden of dementia in a Latin American country using data from a large sample of individuals with dementia or MCI, enrolled from heterogeneous communities; and it will also be the first in Peru to assess changes in health economic outcomes (resource use, costs and HR-QoL) over a 1-year period and examining their association with dementia progression.

Our approach is particularly important in Latin America, where cognitive assessment tools developed in high-income countries may not be appropriate for all population groups.^9^ The high proportion of individuals with low education levels and high socioeconomic disparities impacts neuropsychological test performance, increasing the risk of misdiagnosis.^51 52^ A study showed that lower education is associated with reduced grey-matter volume and weaker brain functional connectivity, which can affect performance on neuropsychological tests^53^; additionally, socioeconomic inequality, environmental pollution and health disparities further influence cognitive assessment results.^54^

This study will also provide a comprehensive estimation of the societal costs related to dementia and how this condition affects the HR-QoL of both participants and study partners (including caregivers). The costing study on dementia conducted by Custodio *et al*^55^ showed that dementia represents substantial costs for families, with monthly expenses approximately 2.5 times the minimum wage. However, this research was conducted in a private healthcare facility in the capital of the country, which is primarily accessed by individuals with a higher economic status compared with the rest of the country. Moreover, in Latin America, studies estimating the economic burden of dementia are scarce, as reported in two systematic reviews.^56 57^

This study has some potential limitations. First, the population is expected to have a high stigma and low knowledge of dementia, which may discourage participation in the study. This challenge is currently being addressed by the IMPACT Salud project through the implementation of communication campaigns to enhance dementia awareness. Second, this study relies on the participation of study partners to answer specific questions. However, we acknowledge that study partners may have different levels of knowledge about the participant, depending on factors such as the amount of time they spend together, among other characteristics. To account for these differences, certain characteristics, such as caregiving hours and family relationship, will be recorded. Third, self-reported information, particularly resource utilisation and out-of-pocket expenses, may be affected by recall bias or reluctance to share information about their expenses. To partially mitigate this, we will provide each participant with a calendar after completing the baseline evaluation; this calendar will have a space for recording monthly resource utilisation and expenditures to improve the accuracy of self-reported data in the follow-up evaluations.

In line with the WHO’s Global Action Plan for Dementia,^58^ addressing dementia as a public health priority requires a comprehensive health system approach. Having an accurate and appropriate strategy for early detection of dementia is crucial not only from a clinical perspective, but also for resource planning at the policy level. This research will provide valuable insights into scalable, technology-based solutions for dementia care in LMICs, with the potential to improve patient outcomes and health system efficiency. Following the completion of this study, the detection tool will be integrated into a sample of primary health centres, enabling task-shifting of cognitive and functional impairment detection to non-specialist providers in underserved regions of Peru.
