# Supplementary material for: Validation and cost-effectiveness of an mHealth tool for cognitive impairment detection in Peru: protocol for the IMPACT Salud observational study
Source: BMJ Open. 2025 Nov 9;15(11):e107142. doi: 10.1136/bmjopen-2025-107142 (PMC12598945; doi:10.1136/bmjopen-2025-107142)
Supplement: Supplementary file 1 [file bmjopen-2025-107142.reviewer_comments.pdf]

PEER REVIEW HISTORY

BMJ Open publishes all reviews undertaken for accepted manuscripts. Reviewers are asked to complete a checklist review form and are provided with free text boxes to elaborate on their assessment. These free text comments are reproduced below.

ARTICLE DETAILS

Title (Provisional)

Validation and cost-effectiveness of an mHealth tool for cognitive impairment detection in Peru: protocol for the IMPACT Salud observational study

Authors

Cardenas, Maria Kathia; Anza-Ramirez, Cecilia; Bernabe-Ortiz, Antonio; Custodio, Nilton; Montesinos, Rosa; Miranda, J. Jaime; Da Re, Marco; Belon-Hercilla, Maria Veronica; Lazo-Porras, Maria; Hawkins, Jemma; Diez-Canseco, Francisco; Moore, Graham; Whiteley, William; Calvo, Rafael; Cuba-Fuentes, Maria Sofia; Landeiro, Filipa Isabel Trigo; Butler, Christopher R.

This article was not externally reviewed at BMJ Open. Protocols that have been independently assessed prior to submission to BMJ Open are usually fast-tracked to publication on the grounds that further substantial changes will not be possible. This independent assessment will usually be external, independent review for both a substantial grant award from a non-commercial or government funder and ethics approval.
